# Mapping the metagenomic landscape: combined shotgun sequencing and quantitative PCR to profile gut metagenome-assembled genomes in marmosets following treatment with a broad-spectrum antibiotic cocktail

**DOI:** 10.1080/19490976.2026.2687925

**Published:** 2026-06-21

**Authors:** Jordan B. Hernandez, Mayowa Abiodun, Shivdeep S. Hayer, Timothy Dickson, Paul Ayayee, Jonathan B. Clayton

**Affiliations:** a Department of Biology, University of Nebraska Omaha, Omaha, Nebraska, United States; b Nebraska Food for Health Center, University of Nebraska-Lincoln, Lincoln, Nebraska, United States; c Callitrichid Research Center, University of Nebraska Omaha, Omaha, Nebraska, United States; d Department of Genetics, Cell Biology and Anatomy, University of Nebraska Medical Center, Omaha, Nebraska, United States; e Department of Biomedical and Translational Sciences, University of South Dakota, Sioux Falls, South Dakota, United States; f Department of Food Science and Technology, University of Nebraska-Lincoln, Lincoln, Nebraska, United States; g Department of Pathology and Microbiology, University of Nebraska Medical Center, Omaha, Nebraska, United States; h Primate Microbiome Project, University of Nebraska-Lincoln, Lincoln, Nebraska, United States

**Keywords:** Amr, resistome, absolute abundance, microbial load, workflow, gut dysbiosis, non-human primate

## Abstract

Broad-spectrum antibiotics are invaluable tools for treating pathogenic infections, but their sustained use can contribute to changes in gut microbiome membership and the emergence of antimicrobial resistance. While these unintended side effects are independently well documented, the relationship between them has seldom been investigated. To address this, we quantified the effects of 28-d antibiotic cocktail exposure on metagenome-assembled genomes and antibiotic resistance genes in common marmosets using a custom whole-genome shotgun sequencing pipeline and quantitative polymerase chain reaction assays. We observed contrasting genus-level reductions in *Bifidobacterium* abundance and *Fusobacterium* growth, both during antibiotic treatment and a 2-week post-treatment period. Total bacterial abundance was not significantly affected by antibiotics, likely due to the presence of antibiotic-resistant opportunists. Genes for vancomycin resistance and multidrug efflux pumps were identified in metagenome-assembled genomes of an unclassified *Sarcina sp*. and *Escherichia coli*, respectively, and were accompanied by increased abundance of these species during treatment. Additionally, we detected 11 dysregulated metagenomic pathways related to carbohydrate metabolism, including 2 pathways relevant to short-chain fatty acid production, following antibiotic exposure. This study provides insights into the species-dependent emergence of antimicrobial resistance mechanisms in non-human primates following antibiotic exposure that could be relevant for antibiotic therapies and resistance management.

## Introduction

Antibiotics are an important tool in modern medicine that can be used to control the otherwise unmitigated growth of pathogenic bacteria, but their administration can have unintended consequences within the gut microbiome by pushing resident gut bacteria from a healthy “eubiotic” state to an unhealthy “dysbiotic” state.^1^ While gut dysbiosis (an imbalance in microbiome composition often characterized by the emergence of harmful bacteria and loss of beneficial bacteria) following antibiotic treatment is a well-documented phenomenon,^2^ the specifics of how the gut microbiota are affected and how the microbiota affects their host in turn are only somewhat understood. Overarching trends such as a drop in alpha diversity, depletion of taxa from the phyla *Bacteroidota*, *Bacillota*, and *Actinomycetota*, and lower levels of short-chain fatty acids (SCFAs) have been reported in multiple studies.^3^ However, antibiotic exposure can also have variable effects depending on the type, dose, and administration route chosen, as well as on specific host factors (e.g., diet, genetics) and the microbial community itself.^3^ For these reasons, continual development of new antimicrobial therapeutics and refinement of existing approaches are crucial endeavors in the face of growing global microbial resistance to antibiotics.^4^ ^,^ ^5^

Antimicrobial resistance (AMR) refers to the ability of bacteria to develop a tolerance to antimicrobial compounds such as disinfectants, antiseptics, or antibiotics, often by directly or indirectly reducing the availability of molecules targeted by the compound.^6^ The mechanisms by which AMR can occur, and those of the antimicrobials themselves, are strikingly diverse^7^ but can be summarized by a core set of microbial genes known collectively as the resistome.^8^ The antibiotic resistome includes all antibiotic resistance genes (ARGs) with a known role in conferring bacterial resistance.^9^ Once acquired, ARGs can be inherited by daughter cells through binary fission (vertical gene transfer) or transmitted to neighboring cells through the process of horizontal gene transfer,^10^ allowing resistance to spread through the entire microbial community. There is a fitness cost of having ARGs in the absence of a particular antibiotic, but persistent overuse of antibiotics can positively select for these genes and facilitate their accumulation,^10^ leading to the creation of so-called “superbugs” that become resistant to nearly all known classes of antibiotics.

In addition to the disease risks posed by AMR,^11^ further antibiotic-related complications can arise if host‒microbiome symbiosis is disrupted. Beyond their roles as digestive agents, nutrient supplementers, and detoxifiers, the microbiota also contribute to host homeostasis by producing modulatory metabolites that can affect a variety of host functions and processes.^12-15^ Thus, antibiotic-induced gut dysbiosis has been associated with numerous health conditions, including bowel diseases,^16^ ^,^ ^17^ obesity,^18^ cancer,^19^ and mental illness.^20^ ^,^ ^21^ Though much progress has been made in understanding the relationship among antibiotics, the microbiota, and host health, research has historically been hampered by a lack of reproducibility and difficulty in translating results from mechanism-driven animal studies (primarily conducted using rodents^2^) to clinically focused human studies.^3^ ^,^ ^22^ Further investigation of antibiotic‒microbiome dynamics with non-human primate (NHP) models can both increase our understanding of the organisms studied (thereby improving their health and longevity for sustained biomedical research) and greatly enhance translational relevance.^23^ However, such studies in NHPs are scarce compared with similar studies in rodents. Extant NHP studies have focused on surveying bacteria from antibiotic-treated subjects^24-39^ or profiling ARGs,^40-50^ but to the best of our knowledge, only five studies have inspected both simultaneously,^51-55^ and only one of those^52^ included subjects actively receiving antibiotics. Consequently, the purpose of this study is to further close the knowledge gap between active antibiotic exposure and its effects on the gut microbiome composition, including ARGs, using a NHP model.

The common marmoset (*Callithrix jacchus*) was chosen as the NHP model species to study because of its widespread use and growing demand across biomedical research fields.^56^ ^,^ ^57^ The marmoset microbiome, in particular, has been investigated within many contexts, including host behavior,^24^ ^,^ ^58^ diet,^59^ ^,^ ^60^ bacterial interactions,^61^ ^,^ ^62^ and intestinal disease,^59^ ^,^ ^63^ owing to the presence of many shared taxa between the core marmoset and human gut microbiomes.^59^ ^,^ ^64^ However, no studies have profiled ARGs in marmosets receiving antibiotics. Two ARGs (*arnA* and *arnB*) were detected in the marmoset gut,^40^though AMR was not the primary focus of that analysis. We have previously published results describing numerous changes (including altered gut bacteria, metabolites, and behavior) in marmosets following 28-d exposure to an antibiotic cocktail.^24^ Taxonomic and functional classification were performed on short-read (150-bp) shotgun sequence data as part of that analysis. However, such short-read classification is less accurate compared to classification of assembled contigs,^65^ and co-assembly can potentially produce more robust functional annotations.^66^ Binning contigs into metagenome-assembled genomes (MAGs) allows additional identification of novel species and genome-resolved functional mapping (including ARGs).^67^ Here, we sought to analyze the extensive shotgun sequence data from our previous study with a custom assembly-based workflow to assess whether (1) the functional potential of the assembled gut metagenome changes in response to broad-spectrum antibiotic exposure; (2) ARGs are present in the marmoset gut microbiome; (3) ARG abundance changes as a result of antibiotics; and (4) bacteria possessing ARGs could feasibly protect themselves from the antibiotics used in this study. Additionally, the release of annotations that were produced from these assessments will be relevant for future studies focused on the impact of antibiotic perturbation on the microbiome.

## Materials and methods

### Study design and sample collection

**Figure 1.**
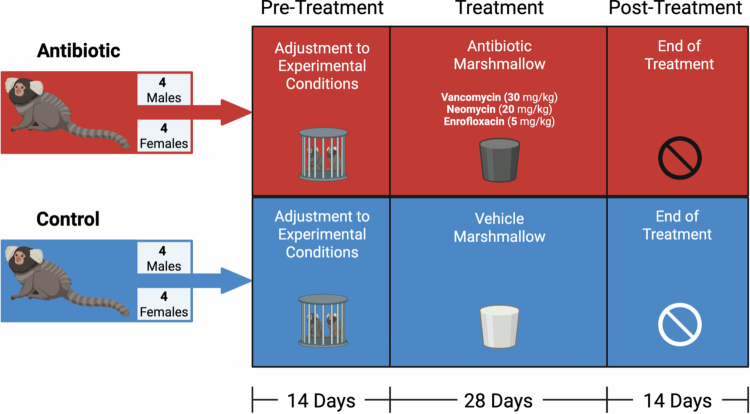
Collection scheme and treatment conditions for collected samples. Marmosets were split into two groups and co-housed in heterosexual pairs. The experiment consisted of three phases and lasted a total of 56 d. In the treatment phase, the antibiotic marmosets received marshmallows containing a broad-spectrum antibiotic cocktail, while control marmosets received vehicle marshmallows. No treatment was administered in the post-treatment phase. Figure created in BioRender.

The samples analyzed in this study were originally collected during a previous study by Hayer et al., 2024.^24^ Because a full description of the sample collection procedures and study design are described there, only a brief overview and accompanying figure are included here to aid the reader in interpreting the results (Figure 1). Sixteen adult marmosets were co-housed into male‒female pairs and divided evenly into two treatment groups (control and antibiotic). The experiment was divided into three phases (pre-treatment, treatment, and post-treatment) and spanned a period of 56 days. During the pre-treatment phase, the marmosets were allowed to adjust to the experimental conditions. During the treatment phase, marshmallows containing a broad-spectrum antibiotic cocktail (vancomycin = 30 mg/kg, neomycin = 20 mg/kg, and enrofloxacin = 5 mg/kg) were administered orally to the antibiotic group, while the control group received vehicle marshmallows instead. The administration of antibiotic/vehicle marshmallows ceased during the post-treatment phase. Fecal samples were collected from every marmoset at approximately the same time every day. The samples were scored by collection personnel using the Bristol scale^68^ and stored at −80 °C. DNA was extracted from a subset of fecal samples and used to perform multiple analyses, including whole-genome shotgun sequencing (*n* = 4 samples/subject), during the previous study. The remaining DNA was stored in covered 96-well plates at −80 °C. This previously extracted DNA was used to perform quantitative polymerase chain reaction (qPCR) analysis (*n* = ~14 samples/subject) in the present study. The selection of samples allowed results to be directly compared between shotgun sequencing and qPCR quantitation. Due to the quantity of extracted DNA that was retained, DNA re-extraction was necessary to quantify *Fusobacterium* abundance in a subset of samples (*n* = 52; Supplementary File 1). Extraction was performed as previously described.^24^

### qPCR assays

The *Bifidobacterium* xylulose-5-phosphate/fructose-6-phosphate phosphoketolase (*xfp*) gene, *Fusobacterium* RNA polymerase beta subunit gene (*rpoB*) gene, and the universal 16S rRNA primer (spanning a 150-bp fragment of the V3-V4 region) were selected for qPCR quantitation. These genomic target regions, as well as the primers designed, were selected owing to their specificity and extensive use as taxonomic tools for the identification and phylogenetic analysis of their respective targets (Table 1). We first verified the presence of these genes in DNA samples via conventional PCR using previously described primers and cycling parameters,^69-71^ followed by the cleaning and sequencing of the selected products. The validated *xfp*, *rpoB*, and 16S rRNA PCR target products were submitted to Integrated DNA Technologies, Inc. (Coralville, Iowa), for designing and synthesizing of custom gBlocks gene fragments. These gBlock gene fragments were 292 bps, 486 bps, and 251 bps for *xfp*, *rpoB*, and 16S rRNA genes, respectively. The gBlock gene fragments were subsequently used as DNA standards for the generation of quantification standard curves for all the qPCR experiments.

**Table 1.** qPCR targets and primer sets used in this study.

Target	Primer Sequence (5ʹ-3ʹ)	Reference
Bifidobacterium (*xfp* gene)	F: ATCTTCGGACCBGAYGAGAC	[70]
	R: CGATVACGTGVACGAAGGAC	
Fusobacterium (*rpoB* gene)	F: GCCTCATTTTHYTDGARTTYCAATT	[71]
	R: ACDACTCTTTCHGCCCATTKHHAT	
Total bacteria (16S rRNA gene)	F: ACTCCTACGGGAGGCAGCAGT	[69]
	R: TATTACCGCGGCTGCTGGC	

The copy number for each quantification target in the fecal DNA samples was calculated using the equation described by Lee et al., 2006.^72^ Absolute quantification of the target genes was performed using a Bio-Rad CFX Connect Real-Time PCR Detection System in combination with the PowerTrack SYBR Green Master Mix (Applied Biosystems, USA; Cat. No. A46109). Reactions were carried out in 96-well non-skirted PCR plates (Fisher Scientific; Cat. No. 14-230-232) and sealed with optical adhesive covers (Applied Biosystems; Cat. No. 4311971). For all the samples and standards, a 20 μL reaction volume was used following the manufacturer’s instructions. Each well contained 10  μL of 2× SYBR Green Master Mix, 1 μL of forward and reverse primers (final concentration: 300 nM each), 6 μL of DNase-free water, and 2 μL of template DNA. The DNA template was obtained from either fecal DNA samples or the gBlock gene fragment DNA standard (all eightfold serial dilutions for standard curve generation). All the reactions were performed in triplicates.

Amplification was performed under the following cycling conditions: initial denaturation at 95 °C for 1 minute, followed by 35 cycles of denaturation at 95 °C for 15 seconds, annealing for 1 minute at 60 °C for *Bifidobacterium* xfp gene, 50 °C for *Fusobacterium* rpoB gene, and 55 °C for 16S rRNA gene for all the bacteria; and an extension at 72 °C for 1 minute for all targets. Standard curves were generated by plotting the cycle threshold values of the gBlock dilutions against the logarithm (log₁₀) of their calculated gene copy numbers.^73^ Melting curves were obtained from 65 °C to 95 °C, with fluorescence measurements taken at every 1 °C increase in temperature. All reactions were carried out in triplicate, along with a non-template control. Cycle threshold values were calculated under default settings for absolute quantification using the software provided with the instrument. Analyses of amplification data, automatic linear regression threshold, and absolute gene copy numbers were calculated using the CFX Manager software.

### Shotgun sequencing and snakemake processing pipeline

**Figure 2.**
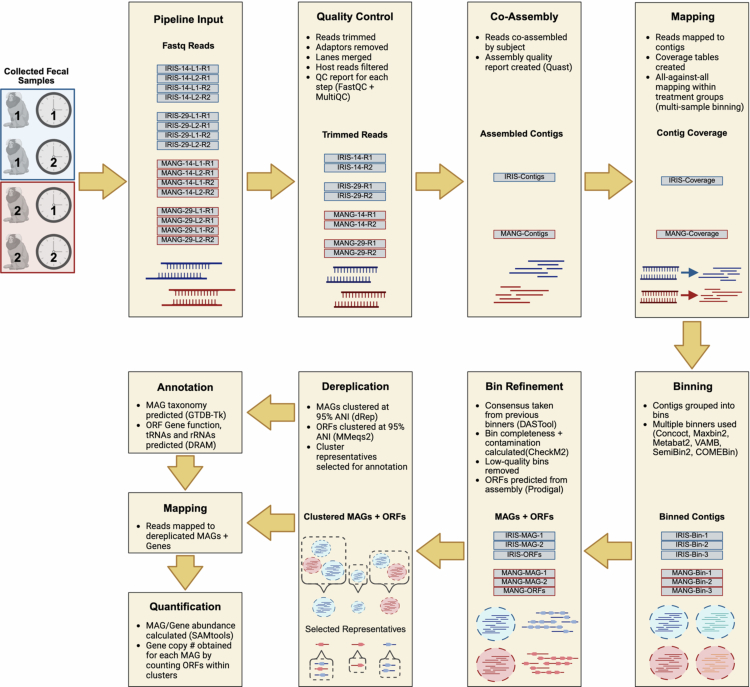
Custom Snakemake pipeline used to process shotgun sequencing samples. A simplified example here shows samples collected from two marmosets (red and blue squares) at two time points (numbered clocks). Each yellow square represents a major step in the pipeline, with corresponding illustrations colored by subject. Following the removal of adaptor and host sequences, the reads are co-assembled and binned at the subject level using a variant of multi-sample binning. Low-quality bins are removed, and the remaining bins are referred to as metagenome-assembled genomes (MAGs). In tandem, open reading frames (ORFs) are predicted from co-assemblies. Following dereplication, MAGs and ORFs are annotated to obtain bacterial taxonomy and KEGG ortholog IDs, respectively. Finally, MAGs/ORFs are quantified from mapped sample reads. Figure created in BioRender.

Shotgun sequence data (2 × 150 bp) were generated on an Illumina NovaSeq 6000 using an S1 flow cell at a sequencing depth of approximately 25 million reads per sample. Bioinformatic processing of the sequenced reads was performed with a custom pipeline implemented in Snakemake^74^ (Figure 2). Trimming of the adaptor sequences was performed with Cutadapt^75^ using a minimum read length of 50 bp. Bowtie2^76^ was used to filter reads aligning to the host marmoset genome (GCA_011100555.2), and processed reads were visually inspected with FastQC and MultiQC.^77^ Following trimming and host filtering, reads were co-assembled into contigs with metaSPAdes^78^ using default settings. These subject-specific co-assemblies were used for all subsequent pipeline steps, including functional pathway quantification and MAG recovery. Gene calling of open reading frames (ORFs) from assembled contigs was performed with Prodigal,^79^ and the nucleotide sequences of the predicted ORFs were clustered at 95% average nucleotide identity (ANI)^80^ ^,^ ^81^ with MMseqs2^82^ in easy-cluster mode at 95% alignment coverage using greedy clustering (cluster-mode 2). Cluster-representative ORFs were then annotated against the KEGG Genes,^83^ CAMPER,^84^ CANT-HYD,^85^ dbCAN,^86^ FeGenie,^87^ MEROPS,^88^ Pfam,^89^ VOGDB,^90^ DRAM methylotrophy and DRAM sulfur databases with DRAM.^91^

Binning of assembled contigs was accomplished using a variation of multi-sample binning whereby reads are mapped to assemblies in an all-against-all manner, with the variation being that pairings were restricted by treatment conditions. This strategy reduced the computational load by reducing the number of read-assembly pairs to map from 1024 (64 read samples × 16 co-assemblies) to 512 (32 read samples × 8 co-assemblies × 2 treatment conditions) while maintaining the superior performance of multi-sample binning compared to conventional single-sample binning.^92^ After mapping reads to assemblies with Bowtie2, contig binning was performed with MaxBin2^93^ (prob_threshold = 0.8), MetaBAT2,^94^ CONCOCT,^95^ and VAMB^96^ using a minimum contig length of 1500 bp, and with SemiBin2^97^ and COMEBin^98^ using default settings. The output from each of these binners was fed into DAS Tool^99^ to create a consensus set of bins, which was further refined using CheckM2^100^ to select near-complete bins (i.e., MAGs) defined as having >90% completeness and <5% contamination but lacking rRNA and tRNA genes.^92^ ^,^ ^101^ ^,^ ^102^ Recovered near-complete MAGs were clustered at 95% ANI and 30% minimum alignment coverage with dRep.^103^ Taxonomy was predicted for cluster-representative MAGs with GTDB-Tk.^104^ A label of “unclassified” was assigned to taxonomic levels left blank by GTDB-Tk, and multiple unclassified taxa within a classified parent level (e.g., two unclassified species within a classified genus) were treated as distinct taxa given that they shared <95% ANI, and this threshold provides sufficient resolution to differentiate between species.^105^ ^,^ ^106^

Reads from each sample were mapped to cluster-representative ORFs and MAGs with Bowtie2, and quantification was performed with the SAMtools^107^ coverage command using the *numreads* column (i.e., the total number of mapped reads) for mapped ORFs and the *meandepth* column (i.e., mean depth of coverage) for mapped MAGs. DRAM annotation files were then parsed with an in-house Python script to extract KEGG ortholog (KO) IDs associated with each ORF and link annotated ORFs to contigs and MAGs. After linking, additional in-house scripts aggregated ORF and MAG coverage files to create an abundance table for each sample. ORF coverage was summed in cases where multiple cluster-representative ORFs were assigned the same KO by DRAM. Gene lengths were obtained with SAMtools idxstats, and gene copy number was obtained by counting the number of ORFs assigned to each cluster. The total gene abundance was then normalized by copy number and converted to transcripts per million (TPM). No such normalization was needed for MAG abundance, because the mean coverage depth already takes transcript length into account.^108^ ^,^ ^109^

### ARG annotation

ARGs were identified for dereplicated ORFs outputted from the shotgun sequencing pipeline with AMRFinderPlus (database version 25-07-16.1) using the --plus option.^110^ ARG abundance was obtained by reusing the ORF-contig-MAG linking file output by the pipeline and joining it with the dereplicated ORF coverage file and AMRFinder output table (which included the ORF ID of each database hit). As with DRAM annotation, ORF coverage was summed in cases where multiple cluster-representative ORFs were assigned the same NCBI protein ID by AMRFinder.

### Differential abundance and diversity testing

Differential abundance and diversity testing were performed by running a combination of R and Python code in a University-hosted Jupyter Lab environment as previously described.^62^ The absolute copy numbers obtained from the qPCR assays were log_10_ transformed to assure normality and homoscedasticity (confirmed using a Shapiro‒Wilk test) and fitted to a linear mixed-effects model with lme4 and lmertest using the following formula:

Abundance~Sex+Treatment_Group*Experiment_Phase+(1| Subject_ID).

The subject was modeled as a random intercept to account for repeated measures, and the main variable of interest was the interaction between the treatment group and the experiment phase. This same formula was used for all other significance testing unless otherwise noted.

The phyloseq package was used to calculate the Shannon alpha diversity index for rarefied MAG counts, and significance was assessed with a mixed-effects model.^24^ Unrarefied MAG count tables were converted to relative abundance and multiplied by the qPCR bacterial load to approximate the absolute species abundance.^111-113^ This qPCR-adjusted abundance was used for all subsequent statistical analyses and visualizations that involve MAG abundance. Bray‒Curtis dissimilarity was chosen as a metric of beta diversity and calculated for species-level MAG abundance and TPM-normalized metagenome abundance. The adonis2 function in vegan^114^ was used to perform PERMANOVA testing for marginal effects across 9999 permutations. The effect of the experiment phase was tested by restricting permutations to stay within each subject, whereas the effects of sex, treatment group, and age were tested simultaneously by permuting freely between subjects.

Differential MAG abundance at the species level was estimated with ANCOM-BC2^115^ using the previously described mixed-effects formula. False discovery rate (FDR) adjustment using the Benjamini‒Hochberg procedure^116^ was performed by adding an additional parameter to the ancombc2 function call. The counts of sample reads mapped to DRAM-annotated genes were TMM normalized with edgeR,^117^ filtered according to the edgeR authors’ recommendations,^118^ and log_2_ + 1 transformed before being fitted to a mixed-effects model. The model was fitted on a per-gene basis and, in cases where fitting was singular (i.e., subject variance was essentially zero), a simpler model with a fixed subject effect was fitted instead.^119^ Post-hoc significance testing was performed with the emmeans package using the formula “revpairwise ~ Treatment_Group | Experiment_Phase” (post-hoc adjustment = False), and the resulting *p*-values were manually adjusted for FDR at 5% using the Benjamini‒Hochberg procedure.^116^

Because the ARGs had a much higher percentage of zeros than the DRAM-annotated genes (Figure S1), a generalized linear model (GLM) was built for each ARG in the unadjusted count matrix using glmmTMB. Each GLM assumed a negative binomial distribution and used the log product of edgeR’s normalization factors (library size and a scaling variable) as an offset^120^ with a log linking function. In cases where fitting failed (indicated by a non-positive definite Hessian, failure to converge, or undefined standard error for the model), the subject was fitted as a fixed effect instead. Filtering and post-hoc significance testing was performed for ARGs in the same manner as with DRAM-annotated genes.

### Pathway enrichment analysis

Pathway enrichment was performed by extracting KO-pathway linking files from the KEGG database (version 2021-01-18) and performing a hypergeometric test (overrepresentation analysis)^121^ against significant KOs identified from metagenome differential abundance analysis. Significant genes annotated with multiple KOs had all corresponding KOs included in the enrichment analysis. The hypergeometric test was conducted with Scipy’s hypergeom module using all KOs in the KEGG database as the background distribution. The resulting table of *p*-values was adjusted for FDR at 5% using the statsmodels module with the Benjamini‒Hochberg procedure^116^ each time the hypergeometric test was performed.

### Correlation analysis and data visualization

Processed shotgun sequencing count tables and sample metadata were imported as objects in phyloseq,^122^ and metagenome count data were imported as objects in edgeR.^117^ The labdsv package^123^ was used to calculate Bray‒Curtis dissimilarity, and Principal Coordinates Analysis (PCoA) plots were generated with vegan and ggplot2.^114^ ^,^ ^124^ Volcano plots for differentially abundant genes were created with ggplot2.^124^ Taxonomic barplots for qPCR-adjusted MAG abundance were created with fantaxtic.^125^ The microbiome R package^126^ was used to calculate the Pearson correlation coefficient between paired shotgun sequencing and qPCR samples, adjust for multiple tests, and generate the resulting heatmaps.

## Results

### Absolute quantification confirms that *Fusobacterium* spp. thrive while *Bifidobacterium* spp. plummet following antibiotic treatment

**Figure 3.**
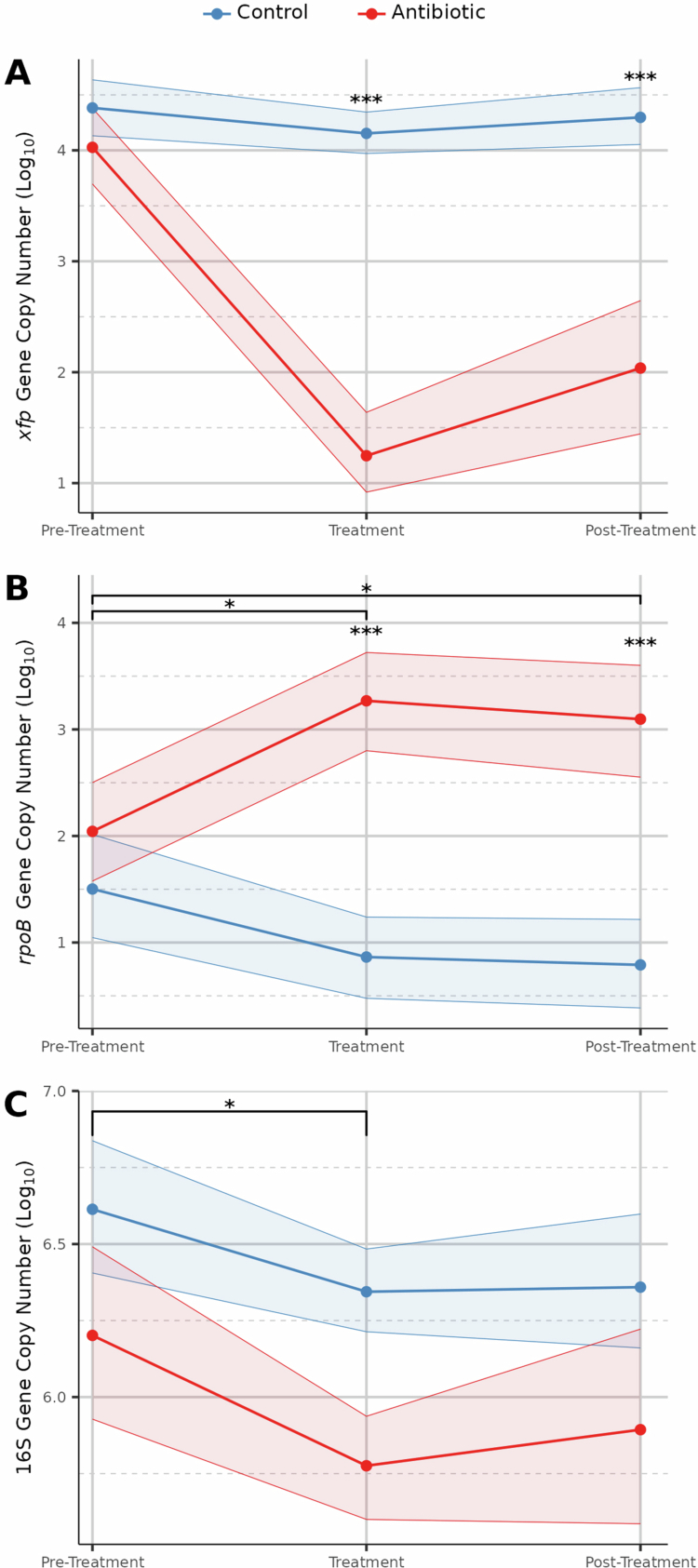
Absolute quantification of *Bifidobacterium*, *Fusobacterium*, and total bacterial load in collected fecal samples. Log_10_ copy number was measured by qPCR using primers that target, (A) the *xfp* gene (unique to the *Bifidobacterium* genus), (B) the *rpoB* gene (unique to the *Fusobacterium* genus), and (C) the 16S rRNA gene (total bacterial load). The colors denote treatment group averages, and the ribbons denote 95% confidence intervals in each experiment phase. Significance is denoted by the number of asterisks and corresponds to *p* < 0.05 (*), *p* < 0.01 (**), and *p* < 0.001 (***). Brackets depict a change between experiment phases with respect to the Pre-treatment phase, while asterisks without brackets depict group‒phase interactions.

After processing the shotgun data with a custom Snakemake pipeline, a total of 83 unique species MAGs (including 62 potentially novel unclassified species) were recovered. Initial plotting of the shotgun sequencing data showed a clear decrease in *Bifidobacterium* relative abundance with a simultaneous increase in *Fusobacterium* relative abundance following antibiotic treatment (Figure S2), mirroring the results from a previous analysis of these data, which used a different shotgun workflow.^24^ To further verify the reproducibility of our results, genus-level *Bifidobacterium* and *Fusobacterium* abundances and total bacterial abundances (bacterial load) were measured in each sample with qPCR. Statistical analysis of the log-transformed qPCR data identified a significant group‒phase interaction in the abundance of *Bifidobacterium*, indicating that this genus was severely depleted in antibiotic-treated marmosets during the treatment (log_10_FC = −2.9; *p* = 3.02 × 10^−12^) and post-treatment (log_10_FC = −2.2; *p* = 1.22 × 10^−6^) phases (Figure 3A). A concurrent *Fusobacterium* bloom during these phases was also observed (Figure 3B), confirming that a genuine shift in the abundance of fecal *Bifidobacterium* and *Fusobacterium* occurred following antibiotic treatment. A significant change in total bacterial load was detected between the Pre-Treatment and Treatment phases, but none of the group‒phase interaction effects estimated from the fitted model were statistically significant, suggesting that the longitudinal trajectory of bacterial load was similar between the antibiotic and control groups (Figure 3C).

The reported observation that antibiotics had no significant effect on total bacterial load was not anticipated. The *Bifidobacterium* biomass that was lost in the antibiotic group was not completely reoccupied by that of *Fusobacterium* during treatment, indicating that other bacterial populations could have expanded as well. Another possibility is that *Fusobacterium* abundance was underestimated by qPCR. This could occur if there were marmoset-specific *Fusobacterium* species that were not amplified by our human-derived primers, or if excessive technical variation was present from DNA extraction of the fecal samples. The latter case is possible, as DNA had to be reextracted from some samples to perform qPCR for *Fusobacterium*. To test this case, DNA from reextracted samples was used to perform additional qPCR for *Bifidobacterium* abundance, and these samples were correlated with *Bifidobacterium* samples from the original extraction (Figure S3). The samples were highly correlated (*r* = 0.63; *p* = 4.28 × 10^−7^), suggesting that technical variation was not a major confounder of measured *Fusobacterium* abundance.

### Normalization of relative abundance data by qPCR-derived 16S rRNA gene abundance correlates well with absolute *Bifidobacterium* abundance

**Figure 4.**
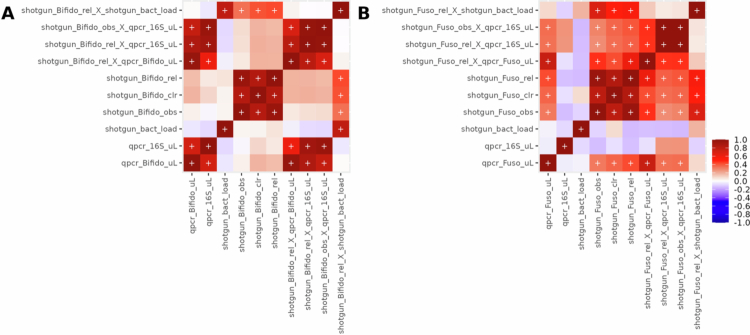
Comparison of various measures of shotgun sequencing and qPCR abundance reveals significant positive correlations. (A) Heatmaps of Pearson correlation coefficients for the abundance of (A) *Bifidobacterium* and (B) *Fusobacterium* at the genus level. Axis labels denote the source of the data (qPCR or shotgun) as well as the type of abundance (rel = relative abundance; obs = observed abundance; clr = centered-log ratio, uL = gene copy number). Labels containing X's denote multiplication (e.g., shotgun relative abundance*qPCR 16S gene abundance). White crosses denote significant correlations (*p* < 0.05) after adjustment at 5% FDR.

To further explore the relationship between *Bifidobacterium*-specific and total bacterial loads, the relative abundances of *Bifidobacterium* spp. MAGs were multiplied by the qPCR bacterial load to approximate absolute abundance at the species level. These “qPCR-adjusted” MAG counts were then summed to the genus level and compared to qPCR-derived absolute genus abundance using the Pearson correlation coefficient. Other measures of shotgun abundance were also included in the correlation analysis for the sake of completeness, including the observed abundance (i.e., read counts), centered log-ratio (CLR) transformation, and an alternative bacterial load metric that can be derived from the ratio of host to non-host reads.^127-130^ Heatmaps showed a significant correlation (*r* = 0.86; *p* = 1.78 × 10^−19^) between qPCR-adjusted *Bifidobacterium* abundance and the known absolute abundance (Figure 4A), indicating that shotgun relative abundance adjusted by qPCR-derived bacterial load closely approximates the true *Bifidobacterium* abundance across most samples. Notably, all the shotgun-derived *Bifidobacterium* abundance measures (CLR-transformed abundance, shotgun-derived bacterial load, relative and observed abundance) correlated poorly with absolute abundance, demonstrating that shotgun sequencing alone cannot accurately approximate absolute abundance.

Similar correlation analysis performed with *Fusobacterium* produced less conclusive results. While the correlation between qPCR-adjusted *Fusobacterium* abundance and the known absolute abundance was still significant across all the samples (*r* = 0.34; *p* = 0.01), the correlations were noticeably weaker (Figure 4B). This could be explained by a disparity between the absolute *Fusobacterium* abundance and qPCR bacterial load, given that these metrics correlated poorly as well. Our correlation analysis between shotgun and qPCR abundance, therefore, suggests that bacterial load adjustment is generally a good estimate of absolute abundance, with the caveat that this estimate may be less accurate for taxa that undergo drastic shifts in abundance with respect to the total biomass (i.e., the bacterium’s abundance changes while the bacterial load does not change or vice versa). Even with this caveat, qPCR adjustment is still preferable to relative abundance because the fecal bacterial load can vary on an order of magnitude even within healthy subjects,^131^ making correlations and differential abundance testing less reliable if not controlled for.^132^ Accordingly, qPCR-adjusted MAG abundance was used for the remainder of this study.

### *Escherichia coli* and several *Fusobacterium* spp. remain enriched 14 d after antibiotic cessation

**Figure 5.**
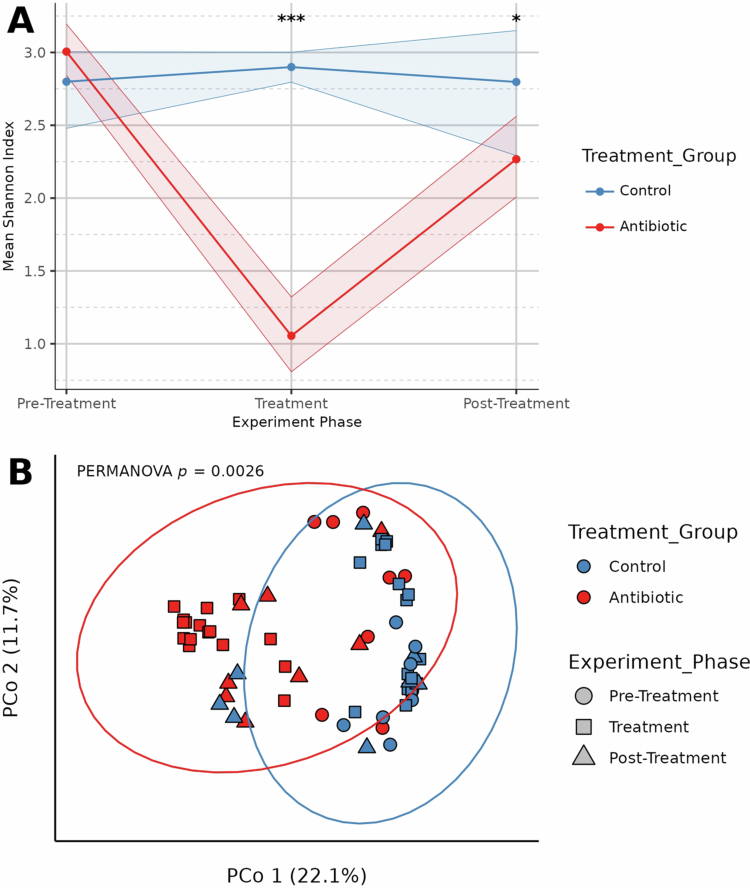
Antibiotics caused a significant change in alpha and beta diversity, as calculated from MAG abundance. (A) A mixed-effects model was fitted to the Shannon diversity index for each sample, and detected a significant group‒phase interactions (*p* < 0.05) during the treatment and post-treatment phases. (B) PERMANOVA was performed on the sample-wise Bray–Curtis dissimilarity matrix of qPCR-adjusted MAG abundance and detected significant differences (*p* < 0.05) between treatment groups and experiment phases.

Alpha and beta diversity were calculated for species MAG abundance using the Shannon index and Bray‒Curtis dissimilarity, respectively. Significance testing with a mixed-effects model confirmed that a significant drop in alpha diversity occurred in the Antibiotic group during the treatment (mean difference = −1.8; *p* = 4.87 × 10^−10^) and post-treatment (mean difference = −0.5; *p* = 0.017) phases (Figure 5A). PERMANOVA analysis of the beta diversity matrix indicated that the treatment group and experiment phase were the main factors affecting abundance, with no significant effects from sex or age (Figure 5B). Differential abundance testing was performed with ANCOM-BC2, and identified 19 species that differed significantly as a product of treatment conditions or experiment phase (Table 2). Of these, an unclassified *Prevotella* was less abundant in the antibiotic group overall compared to the control group, and the remaining species differed as a result of significant group‒phase interactions. During the treatment phase, two unclassified *Collinsella* spp. and 4 *Bifidobacterium* spp. were depleted, while *E. coli*, unclassified *Sarcina*, *Scatoplasma*, *Duodenibacillus*, and four *Fusobacterium* spp. were enriched in the antibiotic group. Two species (*Fusobacterium_A sp900543175* and *E. coli*) remained enriched, while an unclassified *Nanoperiomorbus* and two *Bacteroides* species (*B. faecis* and *B. ovatus*) were uniquely enriched in the antibiotic group during the post-treatment phase.

**Table 2.** Multiple *Bifidobacterium* spp. are adversely affected by antibiotics.

		Fold change		
Genus	Gram type	Abx	Abx: treatment	Abx: post-treatment
Bifidobacterium_hapali	Positive	0.68	−2.46*	−2.01
Bifidobacterium_myosotis	Positive	−0.37	−2.33*	−0.00
Bifidobacterium_unclassified_2	Positive	1.60	−2.20*	0.09
Bifidobacterium_unclassified_4	Positive	0.84	−2.46*	−0.98
Collinsella_unclassified_3	Positive	0.44	−2.17*	0.54
Collinsella_unclassified_4	Positive	0.59	−2.27*	0.59
Sarcina_unclassified	Positive	−1.19	7.24***	4.27
Avilachnospira_unclassified_3	Positive	0.31	−2.13*	−2.30
Scatoplasma_unclassified	Unknown	−0.22	6.53***	3.70
Bacteroides_faecis	Negative	−0.31	1.48	5.09*
Bacteroides_ovatus	Negative	−0.92	0.89	5.91***
Prevotella_unclassified	Negative	−3.04*	0.96	1.78
Fusobacterium_A_mortiferum	Negative	0.04	5.82***	1.51
Fusobacterium_A_sp900543175	Negative	−0.25	7.41***	4.27*
Fusobacterium_A_unclassified	Negative	−0.00	7.06***	4.18
Fusobacterium_B_unclassified	Negative	0.80	5.59**	3.33
Nanoperiomorbus_unclassified	Unknown	1.24	−0.05	3.42*
Duodenibacillus_unclassified	Negative	−1.71	3.87**	1.78
Escherichia_coli	Negative	−3.56	5.62**	6.26*

Species abundance fold changes were calculated between groups with ANCOM-BC. “Abx” refers to groupwise differences between the control and antibiotic groups. “Abx:treatment” and “Abx:post-treatment” refer to significant group‒phase interactions for the Antibiotic group with the treatment and post-treatment phases, respectively. Significance is denoted by the number of asterisks and corresponds to *p* < 0.05 (*), *p* < 0.01 (**), and *p* < 0.001 (***). All *p*-values were adjusted at 5% FDR.

### Antibiotics can have a lingering impact on potential functional pathways related to butanoate metabolism

**Figure 6.**
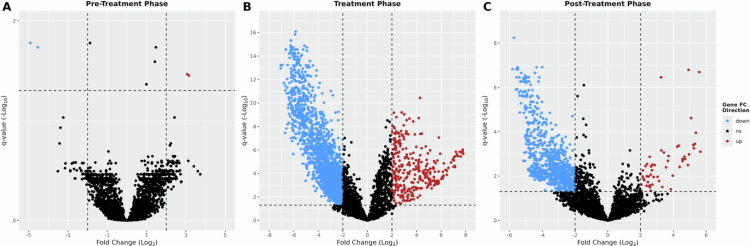
Bacterial gene abundance is altered by antibiotic exposure but trends toward recovery upon cessation. (A‒C) Volcano plots for post-hoc significance of antibiotic group gene abundance compared to controls in the pre-treatment, treatment, and post-treatment phases, respectively. Differentially abundant genes (*p* < 0.05) are colored by their log2-fold change. The dotted lines correspond to the fold change threshold. Negative log_10_ FDR-adjusted *p*-values are shown on the y-axis, and log2-fold changes are shown along the x-axis. All *p*-values were adjusted at 5% FDR.

To explore potential differences in microbiome function as a result of antibiotic treatment, a PCoA plot of metagenome Bray‒Curtis dissimilarity was created (Figure S4). As before, PERMANOVA analysis found significant effects for treatment condition and experiment phase but not for sex or age, indicating that antibiotic treatment also impacts the metagenome. Subsequent fitting with a linear mixed-effects model identified four genes in the Pre-Treatment phase, 1,982 genes in the treatment phase, and 900 genes in the post-treatment phase that were differentially abundant (Figure 6). The vast majority of these genes (1673 and 848 genes in the treatment and post-treatment phases, respectively) were less abundant in the antibiotic group, suggesting a reduction in available functional roles as a result of antibiotic pressure. The fact that this number is approximately halved between treatment and post-treatment further suggests the return of functional roles once pressure is released. Upon closer inspection, 485 genes were significantly depleted in the treatment and post-treatment phases, two genes (K01299 and K17995) were elevated in both, 30 genes switched in direction (either up/down or down/up), and the remaining genes were either not significant or only significant in one phase relative to the controls.

KEGG pathway enrichment analysis was performed on the differentially abundant genes to gain additional insights into the specific functional pathways affected by antibiotics. The analysis revealed 21 pathways that were overrepresented during the treatment phase and 4 that were overrepresented during the post-treatment phase relative to controls (Table 3). Eleven pathways that were affected during the treatment phase were involved in carbohydrate metabolism, and two other pathways were involved in glycan biosynthesis and degradation. Of these, potential modifications to butanoate and propanoate metabolism were particularly intriguing given that these pathways encompass the biosynthesis of the SCFAs butyrate and propionate, respectively. Two altered pathways in the treatment phase (butanoate metabolism and ABC transporters) were also affected in the post-treatment phase, suggesting that these functions had not fully recovered by the end of the study.

**Table 3.** Metagenomic pathways for carbohydrate metabolism are critically impacted by antibiotics.

Enriched KEGG pathway		Treatment phase significant genes			Post-treat phase significant genes		
Subclass	Description	All	Up	Down	All	Up	Down
Cellular community – prokaryotes	Quorum sensing					*	
Membrane transport	ABC transporters	***	*	***	***		***
	Bacterial secretion system				***		***
	Phosphotransferase system (PTS)	***					**
Amino acid metabolism	Histidine metabolism	*		*			
	Phenylalanine, tyrosine, and tryptophan biosynthesis			*			
Carbohydrate metabolism	Amino sugar and nucleotide sugar metabolism	**		**			
	Butanoate metabolism	***	*	*	**		**
	C5-Branched dibasic acid metabolism	*					
	Citrate cycle (TCA cycle)	***		**			
	Fructose and mannose metabolism	***		***			
	Galactose metabolism	*		**			
	Glycolysis/Gluconeogenesis	**					
	Glyoxylate and dicarboxylate metabolism	*					
	Propanoate metabolism	***	***				
	Pyruvate metabolism	**					
	Starch and sucrose metabolism	*		*			
Energy metabolism	Carbon fixation pathways in prokaryotes	***		**			
	Sulfur metabolism	*		*			
Global and overview maps	Biosynthesis of amino acids	***		***			
	Biosynthesis of cofactors	**	*				
	Carbon metabolism	***		**			
Glycan biosynthesis and metabolism	Lipopolysaccharide biosynthesis		**				
	Other glycan degradation	*		**			
	Peptidoglycan biosynthesis	*		**			
Metabolism of cofactors and vitamins	Biotin metabolism		**				
Xenobiotics biodegradation and metabolism	Nitrotoluene degradation		*		*		*

Differentially abundant genes in each experiment phase were partitioned by their log2-fold change value (all = all differentially abundant genes; Down = genes with negative fold change; Up = genes with positive fold change) and inspected for overrepresented pathways using a hypergeometric test. The enrichment significance is denoted by the number of asterisks and corresponds to *p* < 0.05 (*), *p* < 0.01 (**), and *p* < 0.001 (***). All *p*-values were adjusted at 5% FDR.

Additional pathway enrichment analysis performed after partitioning genes by direction (up/down) and phase (treatment/post-treatment) revealed four overrepresented pathways that were not present in the phase-only analysis. In the treatment phase, biotin metabolism and lipopolysaccharide biosynthesis were enriched exclusively in overabundant genes, and the biosynthesis of phenylalanine/tyrosine/tryptophan was exclusive to underabundant genes. Meanwhile, quorum sensing was exclusive to overabundant genes in the post-treatment phase. In total, the analysis of the overabundant genes identified seven enriched pathways during the treatment phase and a single enriched pathway (quorum sensing) during post-treatment compared to controls. Analysis of the underabundant genes found that 16 and four pathways were enriched in the treatment and post-treatment phases, respectively, compared to controls. Together, these results show that antibiotic treatment radically altered microbiome functional potential such that 2 weeks of cessation was not sufficient for a full return to pre-antibiotic baseline.

### Genes for vancomycin, aminoglycoside, and multi-drug resistance identified in recovered MAGs

**Figure 7.**
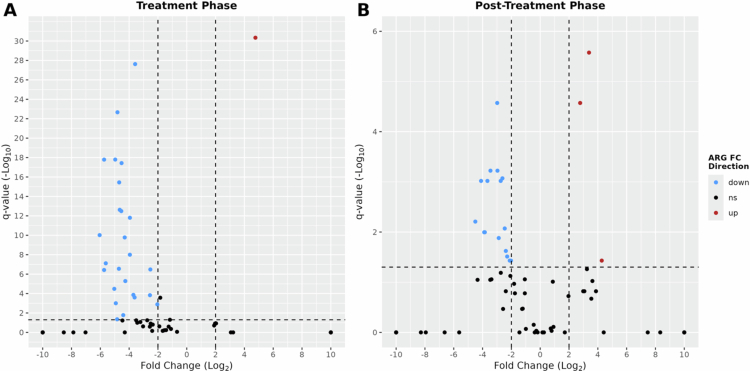
No ARGs relevant to vancomycin, neomycin, or enrofloxacin resistance were enriched as a result of antibiotic administration in the treatment phase. (A and B) Volcano plots for post-hoc significance of antibiotic group ARG abundance compared to controls in the treatment and post-treatment phases, respectively. Differentially abundant ARGs (*p* < 0.05) are colored by their log2-fold change. The dotted lines correspond to the fold change threshold. Negative log_10_ FDR-adjusted *p*-values are shown on the y-axis, and log2-fold change along the x-axis. All *p*-values were adjusted at 5% FDR.

Of the functional pathways affected by antibiotics, ABC transporters were found to be enriched in the treatment phase for both underabundant and overabundant genes relative to those of the controls. This is interesting, as ABC transporters are crucial to cells for housekeeping-related transportation, but they can also contribute to mechanisms of AMR.^6^ To explore this latter area more fully, dereplicated ORF sequences were annotated with AMRFinderPlus to identify ARGs, which have a known role in conferring AMR. Genes from 8 unique classes of AMR were detected (aminoglycoside, beta-lactam, efflux, fosfomycin, glycopeptide, lincosamide/macrolide/streptogramin, phenicol/quinolone, and tetracycline resistance). Annotated ARGs were mapped to sample reads, and differential abundance was calculated using a similar approach to the metagenome data in the previous section (see Materials and Methods for details). Also similar to the metagenome data, most significant ARGs were lower in the antibiotic group during the treatment and post-treatment phases compared to the control group (Figure 7). The only exceptions to this were *blaOXA*, which was overabundant during the treatment phase, and *bla*, *sslE*, and *bexA* during the post-treatment phase. Two of these genes (*bla* and *blaOXA*) are beta-lactamases that confer resistance to beta-lactam antibiotics. The antibiotic cocktail administered in this study included a glycopeptide (vancomycin), an aminoglycoside (neomycin), and a fluoroquinolone (enrofloxacin) but not beta-lactams, so it is unlikely that beta-lactamases provided protection from the antibiotic cocktail. The *sslE* gene is linked to virulence and was included as part of AMRFinder’s “plus” database (as opposed to “core” genes that have a more concrete role in AMR). The final overabundant gene, *bexA*, is another “plus” gene that has been linked to multi-drug resistance, including resistance from fluoroquinolones.^133^ Intrigued by this, we next sought to identify ARGs in the near-complete MAGs that were recovered.

Only ARGs linked to glycopeptide, aminoglycoside, fluoroquinolone, or multi-drug resistance were included in the MAG annotation effort to maximize the likelihood of finding genes that could realistically confer AMR in this study. Even with these constraints, 15 ARGs were identified across ten MAGs (Table 4). In total, MAGs from three species (*E. coli*, *B. faecis*, and *B. ovatus*) possessed genes linked to multi-drug efflux, three others (unclassified *Ellagibacter*, *Megamonas* and *JAGZEG01* sp.) possessed genes linked to aminoglycoside resistance, and four gram-positive Clostridia (unclassified *Sarcina*, *Blautia_A*, *Dorea_D* sp., and *Flavonifractor plautii*) possessed genes linked to vancomycin resistance. Notable standouts from this list include *E. coli*, the two *Bacteroides* spp., and the unclassified *Sarcina*, which were significantly more abundant in the treatment and/or post-treatment phases. *E. coli* in particular possessed four distinct multi-drug efflux genes, living up to its status as a posterchild for multi-drug resistance.^134^ ^,^ ^135^ Surprisingly, *bexA* was not among these genes and was instead present in only the two *Bacteroides* spp. (which did not significantly change their abundance during the treatment phase), suggesting that *bexA*’s enrichment could have originated from horizontal gene transfer to other non-MAG species. Also notable was the detection of multiple *vanR* and *aac(6ʹ)* variants, a potential indicator of antibiotic-induced mutagenesis, which has been documented in response to fluoroquinolone and aminoglycoside exposure.^136^ This is especially true for *F. plautii*, which possessed 4 vancomycin resistance genes, including two unique *vanR* variants.

**Table 4.** ARGs detected in differentially abundant MAGs for *E. coli*, *B. faecis*, *B. ovatus*, and *Sarcina*.

		Multi-Drug Efflux						Vancomycin						Amino-glycoside		
Species	Gram Type	acrF	mdtM	bexA	emrD	emrE	vanR v.1		vanR v.2	vanR v.3	vanR v.4	vanT	vanG		aac(6ʹ) v.1	aac(6ʹ) v.2
Ellagibacter_unclassified	Positive	0	0	0	0	0	0		0	0	0	0	0		1	0
JAGZEG01_unclassified	Unknown	0	0	0	0	0	0		0	0	0	0	0		0	3
Sarcina_unclassified	Positive	0	0	0	0	0	0		0	0	1	0	0		0	0
Blautia_A_unclassified	Positive	0	0	0	0	0	2		0	0	0	0	0		0	0
Dorea_D_unclassified	Positive	0	0	0	0	0	0		0	1	0	0	0		0	0
Flavonifractor_plautii	Positive	0	0	0	0	0	0		2	0	2	3	3		0	0
Megamonas_unclassified_5	Negative	0	0	0	0	0	0		0	0	0	0	0		2	0
Bacteroides_ faecis	Negative	0	0	1	0	0	0		0	0	0	0	0		0	0
Bacteroides_ovatus	Negative	0	0	9	0	0	0		0	0	0	0	0		0	0
Escherichia_coli	Negative	3	2	0	2	2	0		0	0	0	0	0		0	0

Table depicting ARG copy numbers for each MAG. Copy numbers were obtained by summing the number of annotated ORFs within each dereplicated MAG cluster and should therefore be interpreted as the total ARG count across strains, which is not necessarily representative of any one strain in particular. ARGs with multiple variants (which correspond to unique NCBI protein IDs) are numbered with the suffix “v.*n*”.

### Marmosets exhibit looser stool consistency after treatment with antibiotics

When fecal samples were collected during the study by Hayer et al., 2024,^24^ they were scored by collection personnel using the Bristol scale.^68^ This scale rates stool by firmness on a numeric scale from 1–7, with 1 indicating hard, dry stool (i.e., constipation) and seven indicating completely liquid stool (i.e., diarrhea). To determine whether antibiotic-induced dysbiosis had any effect on host wellbeing, the recorded Bristol score associated with each qPCR sample was compared between groups using a linear mixed-effects model. The model identified a significant change in stool consistency during the treatment (*p* = 0.045) and post-treatment (*p* = 0.026) phases compared to the pre-treatment phase (Figure S5). A significant group‒phase interaction further revealed that marmosets in the antibiotic group had a higher overall Bristol stool score compared to the control group during the treatment phase (mean difference = 0.7; *p* = 0.004). The Bristol scale was used to ensure the animals’ safety in the case of prolonged, severe diarrhea, which warranted the removal of an animal from the study. Although transient loose stool was observed in some animals during antibiotic administration, no such removal was necessary.

## Discussion

This is the first study profiling ARGs in marmosets treated with antibiotics. The analysis was enabled by a custom Snakemake pipeline that simultaneously annotated ORFs at the assembly level and taxonomy at the MAG level, extending functional analysis to include genes present in the metagenome but not in the MAGs themselves. Further specificity for unclassified species was achieved by clustering MAGs at 95% ANI—a threshold used to distinguish species within a genus^105^—and by using the total bacterial load to obtain an approximation of absolute microbial abundance.^111-113^ These extra measures allowed 12 unclassified species spanning six phyla to be identified as differentially abundant in marmosets treated with antibiotics. Other notable discoveries from this study include the antibiotic-induced dysregulation of metagenomic pathways for butanoate metabolism and the identification of ARGs in recovered species MAGs.

### How does the functional potential of the assembled gut metagenome change in response to broad-spectrum antibiotic exposure?

The marmoset gut microbiome possesses a plethora of functional pathways for processing carbohydrates and amino acids,^60^ ^,^ ^137^ ^,^ ^138^ suggesting that microbially mediated degradation of host-indigestible fibers is crucial for the health of these primates. Thus, the determined dysregulation of nearly every aspect of carbohydrate metabolism in the antibiotic group relative to controls—from glycolysis and pyruvate to the citrate cycle and SCFAs—during treatment (Table 3) may indicate that depleted bacteria contributed significantly to those pathways. Furthermore, normalizing the relative abundance data to the qPCR-derived bacterial load indicates that even non-significant reductions in species abundance are genuine and not merely the byproduct of more abundant taxa soaking up extra reads (i.e., the problem of compositionality.^139^) From these observations, then, it can be ascertained that bacteria that exhibited the most noticeable depletion from antibiotics (e.g., *Bifidobacterium*; Figure 3) are the greatest contributors to dietary fiber degradation and SCFA biosynthesis in eubiotic marmosets. Overabundant genes in the treatment phase being enriched for butanoate and propanoate metabolism further suggests that alternative bacteria fill these rolls during antibiotic treatment. A marmoset metatranscriptomic study found that one biotin gene and two ARGs were upregulated in cecum and transverse colon samples compared to feces, ^40^ suggesting that antibiotic-resistant bacteria may reside there and supporting an enrichment of biotin metabolism genes during treatment (Table 3). The subsequent trend toward metagenome and microbiome recovery in the post-treatment phase could be due to a significant increase in quorum-sensing genes that trigger microbial growth signals and could also be related to the concomitant spike in *B. faecis* and *B. ovatus*, which are associated with gut eubiosis in humans.^140^ ^,^ ^141^ However, further studies that incorporate gene expression are required to offer greater insights into the mechanisms driving gut microbiome membership and the emergence of antimicrobial resistance in model organisms.

### What ARGs are present in the marmoset gut microbiome?

Intriguingly, all but one of the significantly depleted bacteria during the treatment phase were gram-positive *Actinomycetota*, and all but one enriched species with known gram reactivity was gram-negative (Table 2), suggesting that gram-negative bacteria could adapt more easily to the broad-spectrum antibiotic cocktail. This is further supported by the pathway enrichment analysis, which showed that the biosynthesis of peptidoglycan (a key component of cell walls that are thicker in gram-positive bacteria) was impaired, while biosynthesis of lipopolysaccharides (pro-inflammatory components of gram-negative outer membranes) was amplified in the antibiotic group during the treatment phase (Table 3). Of the antibiotics in our cocktail, vancomycin is most effective against gram-positive bacteria, neomycin is most effective against gram-negative aerobes, and enrofloxacin (along with its degradation product ciprofloxacin) is effective against many gram-negative and some gram-positive bacteria, but is less effective against aerobes.^142^ ^,^ ^143^ Antibiotic selectivity could therefore help gram-negative anaerobes such as *Fusobacterium* establish a foothold during the treatment phase.

Further selectivity insights can be gleaned from the distribution of ARGs identified in recovered MAGs. Indeed, out of 33 MAGs with observed ARGs, 26 were recovered from known or likely gram-negative species, and only four of those possessed ARGs relevant to the antibiotic cocktail used in this study (i.e., ARGs related to multi-drug efflux or those that could confer resistance to vancomycin, neomycin, or enrofloxacin). This number is comparable to that of the five gram-positive MAGs with cocktail-relevant ARGs (four excluding an *Ellagibacter* sp. with a likely superfluous aminoglycoside ARG) (Table 4) and suggests that there was less selective pressure for gram-negative bacteria to acquire protective ARGs. Even if this notion is true, however, the mechanism by which *Fusobacterium* and *Duodenibacillus* spiked relative to other gram-negative anaerobes during the treatment phase is unknown. One possibility is that resistance can be acquired through an indirect mechanism such as microbial biofilms, which can contribute to AMR^144^ and are known to be formed by various *Fusobacterium* spp.^145^. Another explanation is the existence of undocumented AMR mechanisms, either through acquired mutations^136^ or repurposing existing genes. The presence of ARGs across eight unique antibiotic classes in the marmoset gut metagenome, as demonstrated here, offers a strong incentive for future studies to test antibiotic resistance *in vitro* using cultured isolates to better understand potential species selectivity and resistance mechanisms that occur in response to broad-spectrum antibiotic exposure.

### How does the abundance of those ARGs change as a result of antibiotics?

The majority of differentially abundant ARGs were depleted in the antibiotic group during the treatment and post-treatment phases. This makes sense when considering that most of the detected ARGs conferred resistance to antibiotics that were not used in this study. However, there were instances of cocktail-irrelevant ARGs (particularly beta-lactamases) being overabundant and cocktail-relevant ARGs (aminoglycoside and vancomycin resistance genes) being depleted as well. The former scenario likely represents bacteria such as *Fusobacterium* that continue propagating under antibiotic pressure despite possessing cocktail-irrelevant ARGs, potentially through alternative means of resistance that remain unidentified. The latter scenario is more interesting, as it could result from several causes. One explanation could be bacteria that possess incomplete resistance machinery, such as only acquiring a single gene from a two-component system. The *vanR* gene belongs to the VanS/VanR two-component system^146^ and was depleted during the treatment phase, while the *vanS* gene was completely undetected in this analysis. Another explanation could be functionally inert genes that were acquired only as fragments. Yet, another explanation could be bacteria that were resistant to one of the antibiotics in the cocktail but were susceptible to another. The ambiguity arising from many alternative interpretations reflects a key limitation of studies that only do community-level ARG profiling. The MAGs recovered during this analysis can bypass that limitation by allowing genome-resolved identification of ARGs and quantification of species abundance to further confirm resistance.

### Which bacteria possess ARGs that could feasibly protect them from the antibiotics used in this study?

*E. coli*, *B. faecis*, and *B. ovatus* are gram-negative species that exhibited enriched abundance and possessed multi-drug efflux ARGs. The only gram-positive species that was enriched relative to the total bacterial population in the treatment phase was an unclassified *Sarcina* that possessed the *vanR* gene. The genus *Sarcina* (also known as *Clostridium_sensu_stricto_1* ^147^) was reported as a potential keystone genus in the gut of healthy marmosets,^62^ as it was deemed “influential” owing to its associations with many bile acids and amino acids in a bacteria‒metabolite association network graph. It was also enriched in marmosets with intestinal strictures,^148^ with one *Clostridium_sensu_stricto_1 species* (*C. perfringens*) being posited as a causative agent. *Clostridium* spp. are known endospore formers and have demonstrated antibiotic resistance,^27^ ^,^ ^149^ including resistance to vancomycin.^150^ Similar enrichment of *Clostridium* spp., along with *E. coli* and *Fusobacterium nucleatum*, was reported in humans after 4-d treatment with a broad-spectrum cocktail of meropenem, vancomycin, and gentamicin.^151^ It is therefore highly likely that the unclassified *Sarcina* species discovered here is a vancomycin-resistant opportunist that could fill previously occupied niches during treatment and potentially encourage the growth of other antibiotic-resistant taxa.

The observed drop in *Bifidobacterium* spp. and the corresponding rise in *Fusobacterium* spp. during antibiotic treatment supports the previous analysis of these data^24^ and agrees with other studies^59^ ^,^ ^60^ ^,^ ^152^ ^,^ ^153^ by showing that the gut *Bifidobacterium* population in marmosets is extremely fragile and is easily displaced by other taxa in the event of major gut disturbances. However, its gradual recovery in the post-treatment phase showed that *Bifidobacterium* was also resilient to antibiotic perturbation for at least a period of 14 days. This finding lies in stark contrast to a previously mentioned human study where an undisclosed number of *Bifidobacterium* spp. were completely eradicated after 4 days of broad-spectrum antibiotics.^151^ Whether the repopulation of *Bifidobacterium* documented here was facilitated by the arrival of new members through host ingestion or instead occurred through the recovery of a surviving remnant colony is unclear, but the dearth of cocktail-relevant ARGs in *Bifidobacterium* MAGs produced during this study suggests that the probability of any *Bifidobacterium* spp. withstanding a 28-d antibiotic cocktail is low.

### Is shotgun sequencing-derived abundance a good approximation of actual abundance?

The shotgun sequence-derived genus abundance correlated weakly with qPCR-derived bacterial genus abundance. This was true for observed, relative, and CLR-transformed abundance, as well as for relative abundance adjusted by shotgun-derived microbial load. Similar results were obtained when replicating our correlation analysis with 16S amplicon sequence data (Figure S6), indicating that the problem is not unique to our MAG quantification approach. This fundamental disparity between measurements was corrected only after adjusting by qPCR-derived total bacterial load. While a full discussion of absolute quantitation methods is beyond the scope of this analysis, other studies have reported a similar disparity between high-throughput sequencing abundance and absolute abundance using qPCR^139^ ^,^ ^154^ ^,^ ^155^ or other methods such as spike-ins, digital PCR, and flow cytometry.^112^ ^,^ ^131^ ^,^ ^155^ ^,^ ^156^ Thus, our results corroborate the consensus that microbial abundance estimates derived from high-throughput sequencing alone are not an accurate reflection of true abundance and lower alpha diversity does not necessarily correspond to lower bacterial load. Successful rectification of this limitation (possibly through incorporation of absolute abundance measures) is imperative to overcome the reproducibility issues^157^ that frequently plague microbiome high-throughput sequencing analyses.

### Perspectives, conclusions, and future directions

Together, the results of this study show that antibiotics induce the growth of antibiotic-resistant *E. coli* and *Clostridium* at the expense of *Bifidobacterium* spp., and they may alter gut microbiome function in marmosets by reducing the availability of metabolic niches that involve amino acids, sulfur, and especially carbohydrates. Altered butanoate metabolism could potentially affect butyrate synthesis and bacterial synthesis/degradation of γ-aminobutyric acid (GABA),^158^ while bacterial phenylalanine/tyrosine/tryptophan metabolism can include pathways for the synthesis/degradation of dopamine and serotonin.^159^ Two of these neurotransmitters (GABA and serotonin), as well as the SCFA propionate, were previously demonstrated to decrease, while phenylalanine increased, in the antibiotic group during the treatment phase,^24^ an indicator of possible bacterial involvement in these pathways. Indeed, our alpha and beta diversity results, together with significant changes in the abundance of *Bifidobacterium* spp., *Fusobacterium* spp., a *Clostridium* sp., and *B. ovatus*, demonstrated remarkable overlap with the previous analysis of these data. There was also a shared trend of dysregulated metagenome pathways that were involved in the metabolism of amino acids, carbohydrates, and cofactors. Such overlap despite using an almost entirely different analytical methodology, can be seen as a consensus approach^157^ that increases confidence in the validity of our findings.

To our knowledge, this is the first study to quantify the effects of broad-spectrum antibiotics on total fecal bacterial biomass. A higher mucosal bacterial load has been reported in patients with dysbiosis-related intestinal diseases,^160^ and the microbial load has been predicted by machine learning to be associated with numerous diseases,^161^ making the inclusion of absolute quantitation necessary for new microbiome studies. While ORFs and MAGs were clustered at 95% ANI to reduce redundancy,^80^ ^,^ ^162^ ^,^ ^163^ we acknowledge that clustering at the amino acid level is also common^164^ ^,^ ^165^ and that there is no universally agreed upon identity threshold or clustering method to use for metagenomic functional profiling.^163^ ^,^ ^166^ We also acknowledge that qPCR-adjusted abundance could include some variability because of species having multiple copies of the 16S gene.^139^ The antibiotics used in this study do not cross the blood‒brain barrier and were chosen for their low gut absorption^24^ with the goal of inducing drastic, minimally selective gut dysbiosis beyond a reasonable doubt. The administration of a three-drug antibiotic cocktail is comparable to many rodent studies^2^ but might not be directly translational to the short-term clinical use of individual antimicrobials. Further studies using individual antimicrobials that mimic clinical use can be a logical next step in our experiments, wherein the model of multiple, broad-spectrum antimicrobials can serve as a positive control. Another logical next step is to conduct pan-genomic analysis with MAGs^167^ to characterize gene transfer events between species. We also plan to construct a network graph with species abundance data and genome-resolved metabolic pathways to identify putative connections between microbiome composition and function, followed by validation with gene expression assays and/or stable isotope analysis. The findings outlined here serve as a crucial first step in achieving those goals and can be used as a reference for researchers who are interested in translationally focused AMR or metabolic modeling.

## Ethical approval

All procedures conformed to guidelines established by the U.S. National Institutes of Health- U.S. (K01OD030514) and have been approved by the University of Nebraska at Omaha’s Institutional Animal Care and Use Committee (protocol #21-001-08-FC).

## Supplementary Material

Figure S5.jpg Figure S5.jpg

Figure S6.jpg Figure S6.jpg

Figure S2.jpg Figure S2.jpg

Supplementary File 1.xlsx Supplementary File 1.xlsx

Figure S1.jpg Figure S1.jpg

Figure S3.jpg Figure S3.jpg

Figure S4.jpg Figure S4.jpg

Supplementary Material Supplementary figure caption.docx

## Data Availability

The raw shotgun sequence reads, host-aligned reads, MAGs, and functional annotations reported in this paper have been deposited at EMBL-EBI under accession number PRJEB107655. The custom shotgun sequencing pipeline is available at the following URL: https://github.com/clayton-lab/metaGnosis (DOI: 10.5281/zenodo.17715736). Additional software, including the computer code and Conda environment used after shotgun preprocessing, is available at GitHub at https://github.com/clayton-lab/marm_metagenome_manuscript.
