# Supplementary figures and images for: Mapping the metagenomic landscape: combined shotgun sequencing and quantitative PCR to profile gut metagenome-assembled genomes in marmosets following treatment with a broad-spectrum antibiotic cocktail
Source: Gut Microbes. 2026 Jun 21;18(1):2687925. doi: 10.1080/19490976.2026.2687925 (PMC13285596; doi:10.1080/19490976.2026.2687925)

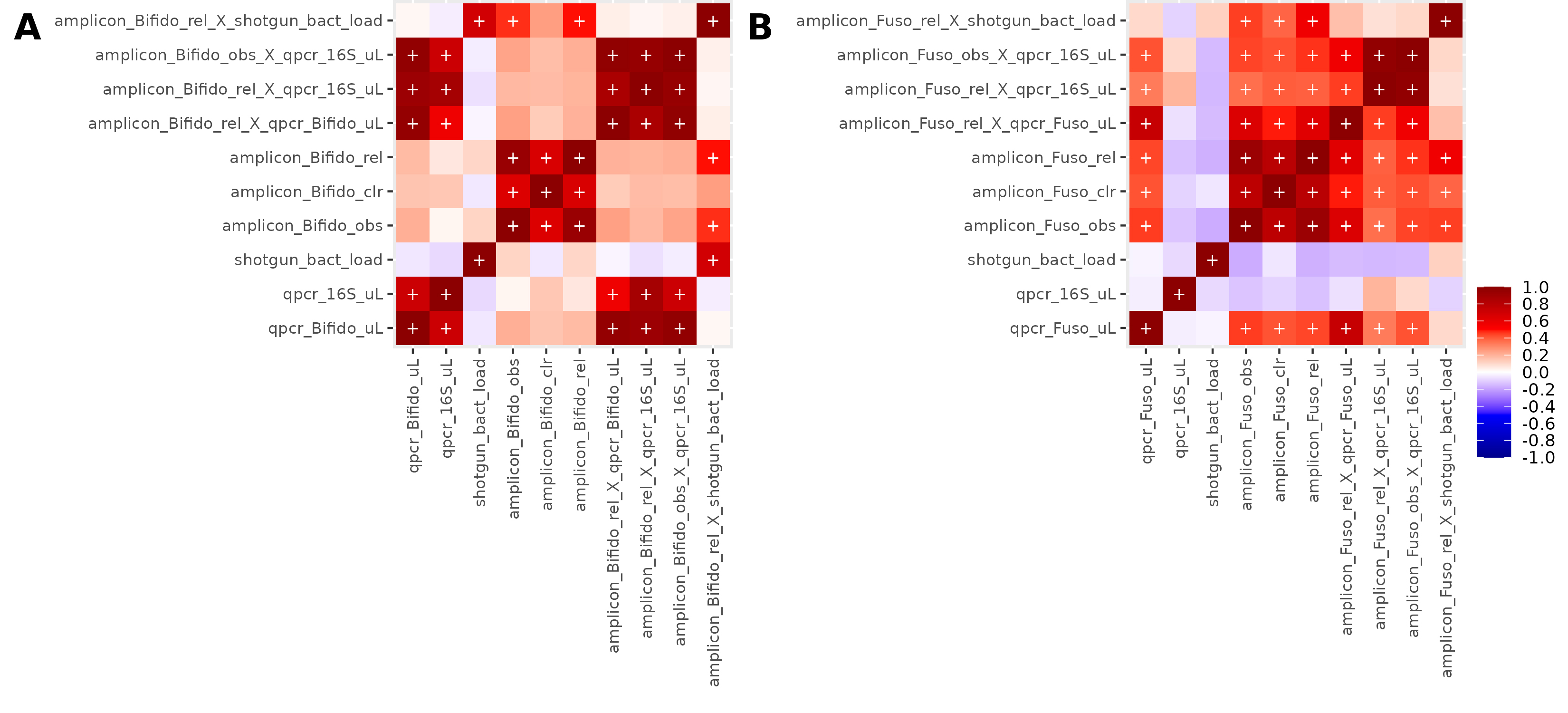

Supplement: Figure S5.jpg [file KGMI_A_2687925_SM1905.jpg]

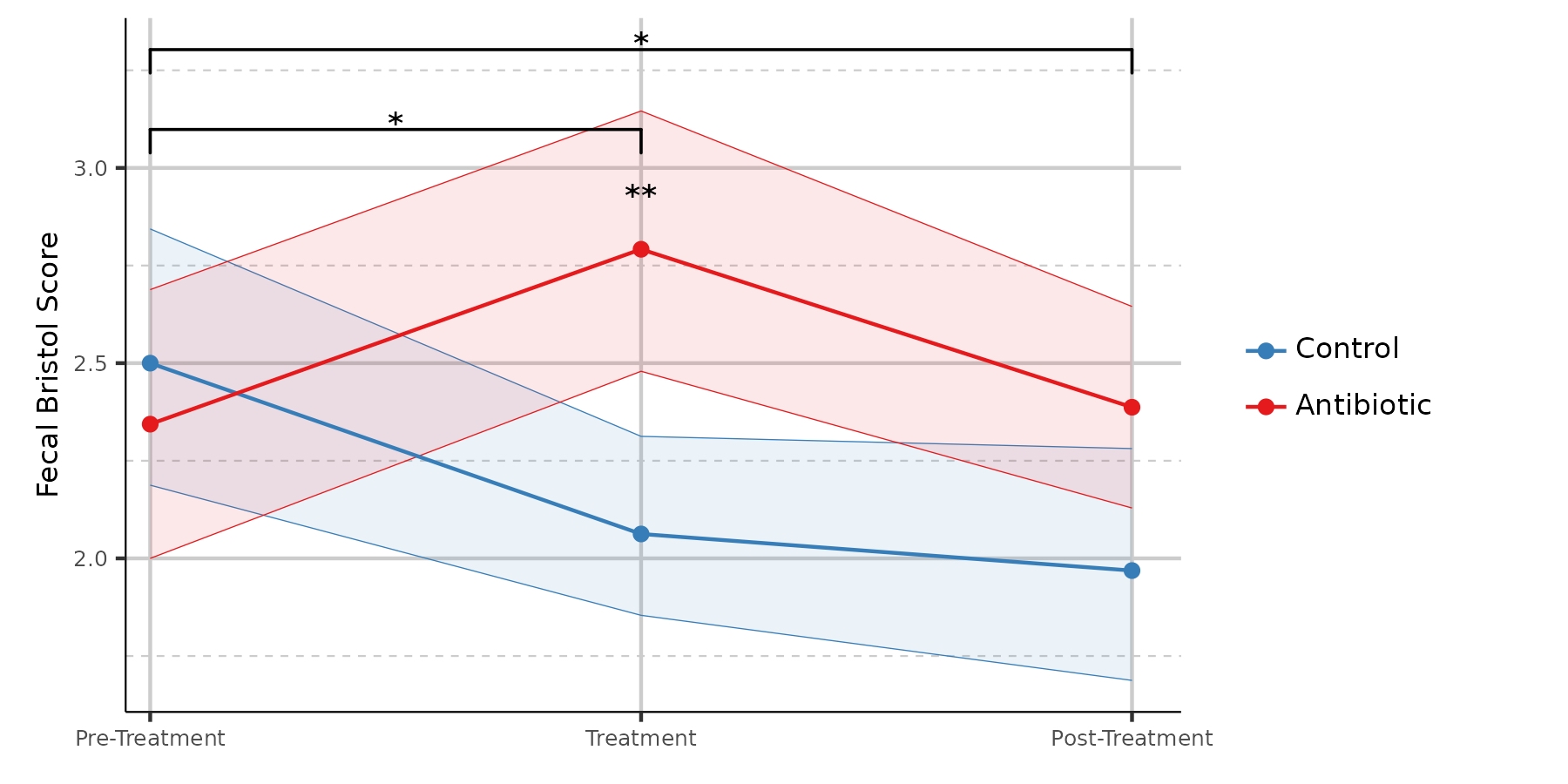

Supplement: Figure S6.jpg [file KGMI_A_2687925_SM1904.jpg]

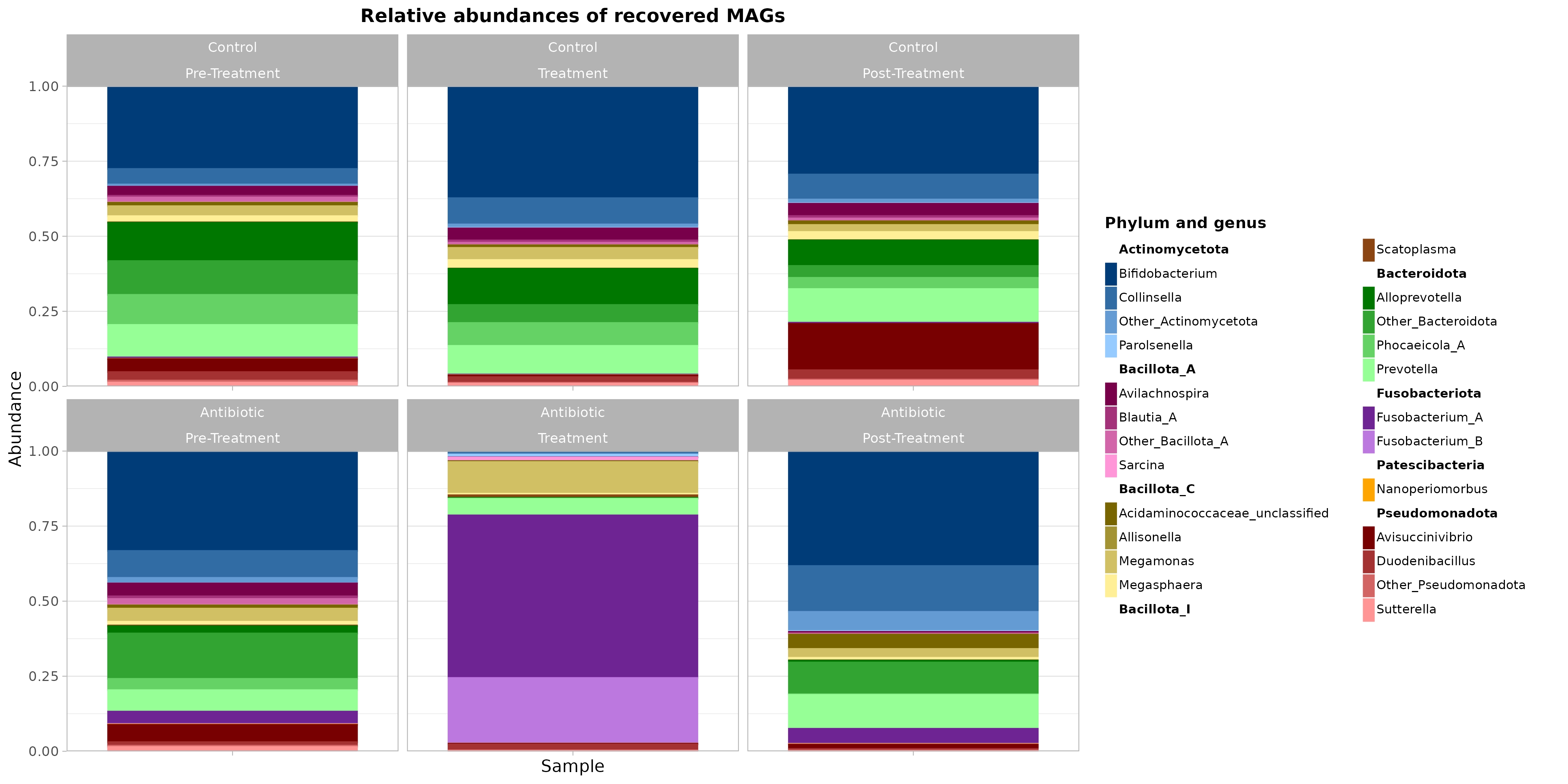

Supplement: Figure S2.jpg [file KGMI_A_2687925_SM1910.jpg]

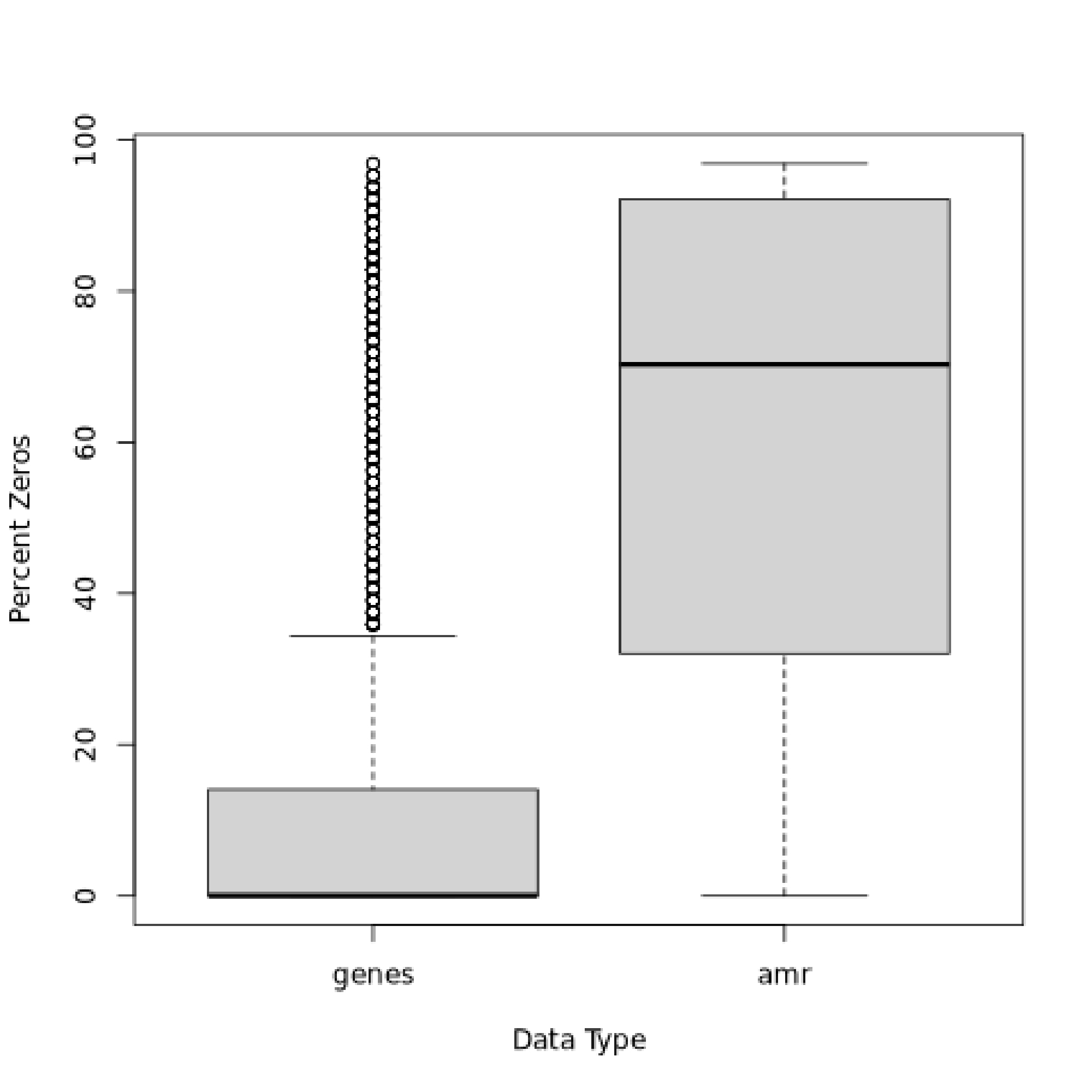

Supplement: Figure S1.jpg [file KGMI_A_2687925_SM1907.jpg]

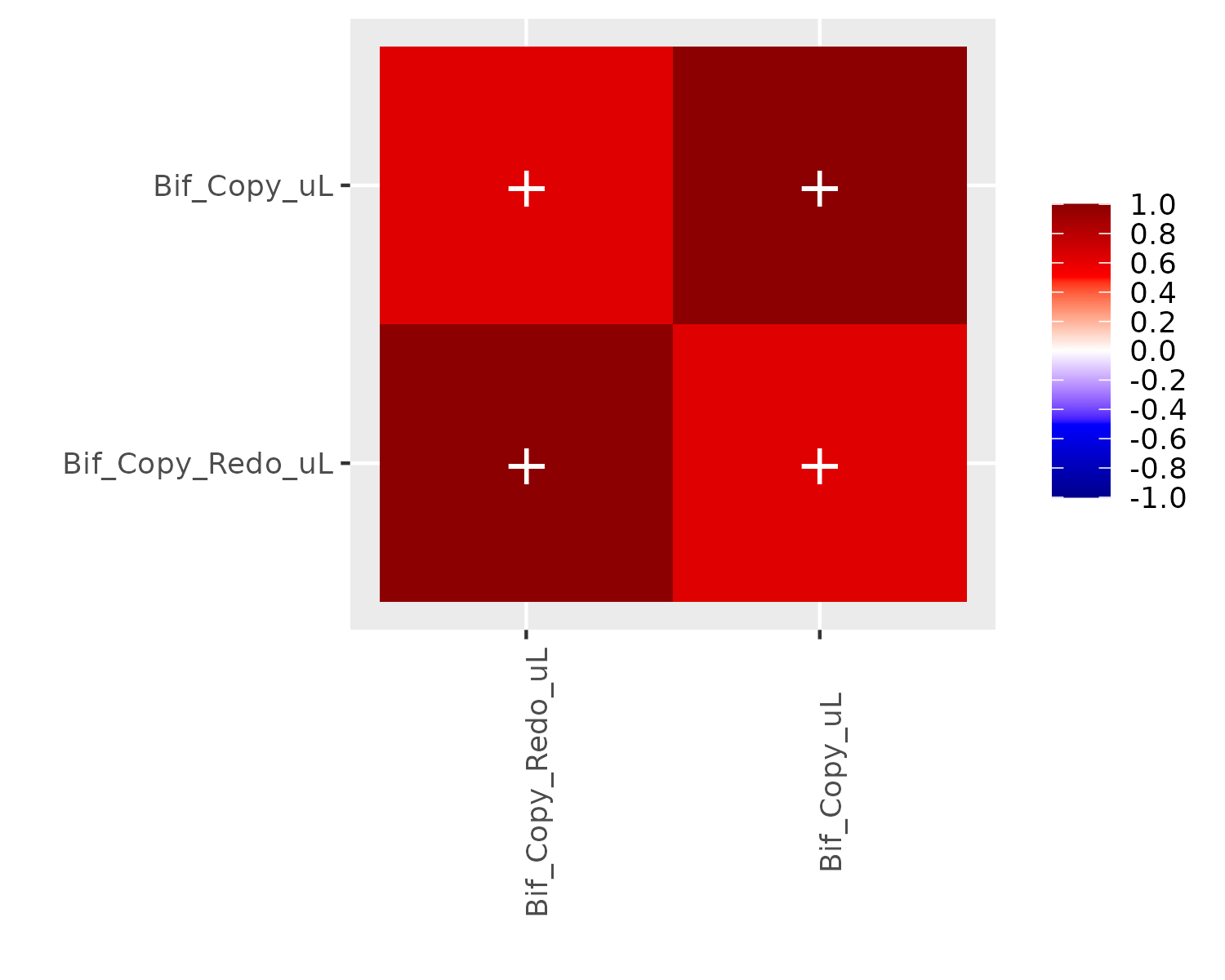

Supplement: Figure S3.jpg [file KGMI_A_2687925_SM1908.jpg]

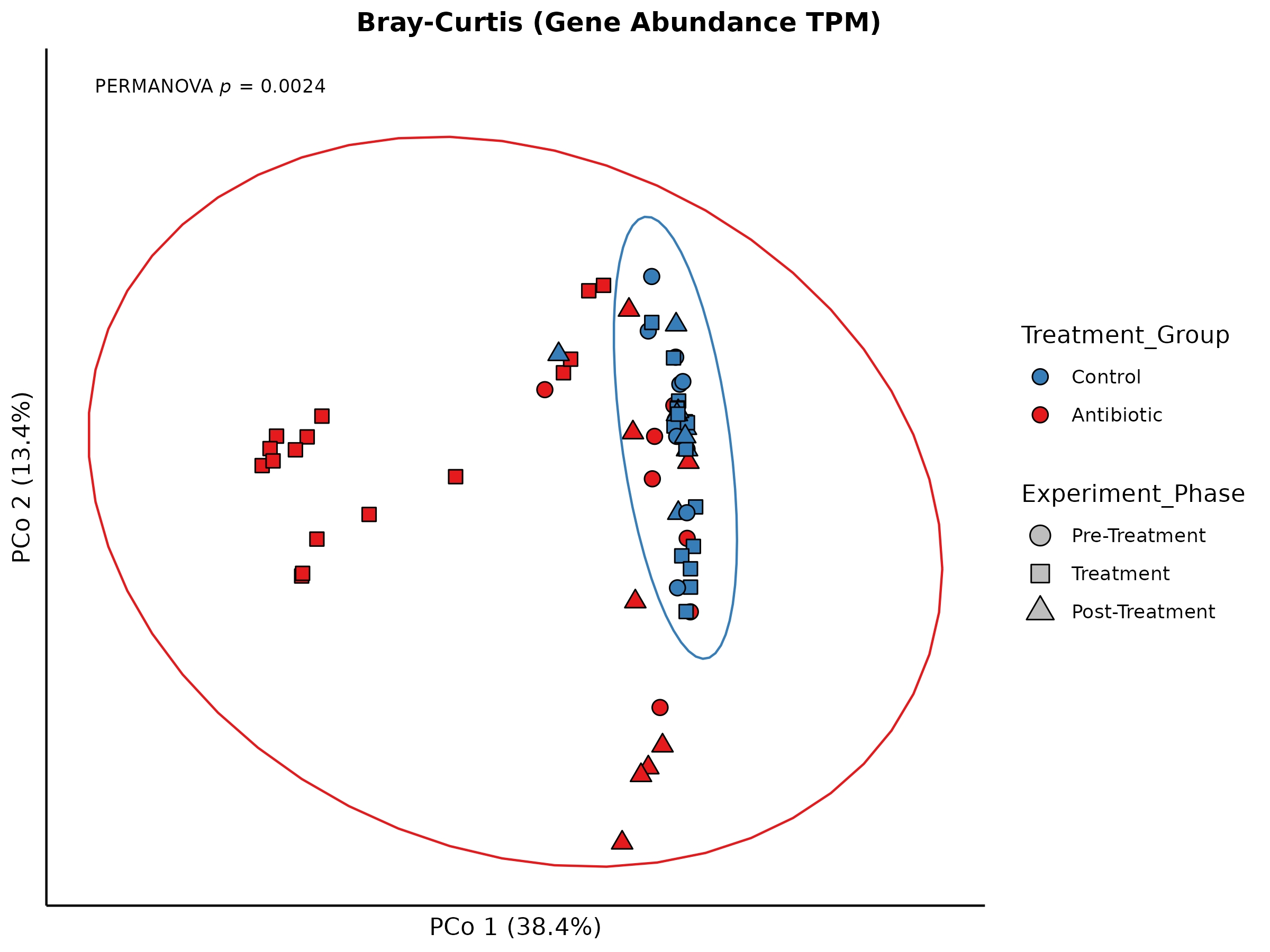

Supplement: Figure S4.jpg [file KGMI_A_2687925_SM1909.jpg]
