# Supplementary material for: Mapping the metagenomic landscape: combined shotgun sequencing and quantitative PCR to profile gut metagenome-assembled genomes in marmosets following treatment with a broad-spectrum antibiotic cocktail
Source: Gut Microbes. 2026 Jun 21;18(1):2687925. doi: 10.1080/19490976.2026.2687925 (PMC13285596; doi:10.1080/19490976.2026.2687925)
Supplement: Supplementary Material — Supplementary figure caption.docx [file KGMI_A_2687925_SM4253.docx]

**Figure S1: Sample prevalence of database-resolved shotgun sequencing genes.** Box-and-whisker plot depicting the percentage of samples for which a gene was not present (i.e., prevalence) for each gene in the DRAM-annotated (left box) or AMRFinder-annotated (right box) gene sets, respectively. Median prevalence is depicted by horizontal lines; boxes span from the first quartile to the third quartile of the data; whiskers depict an interval of 1.5 multiplied by the interquartile range. Data points that fall outside this interval are depicted by circles.

**Figure S2: *Bifidobacterium* is displaced by *Fusobacterium* in marmosets treated with antibiotics.**

Bar plot of MAG relative abundance showing the 3 most abundant genera from every phylum, summed across samples. Control marmosets are dominated by bacteria from the phyla of *Actinomycetota* and *Bacteroidota*, while marmosets treated with antibiotics are dominated by *Fusobacteriota* during the Treatment phase. Gradual recovery toward pre-antibiotic baseline can be seen in the Post-Treatment phase.

**Figure S3: Correlation analysis of *Bifidobacterium* abundance shows minimal variation from DNA extraction.**

*Bifidobacterium* abundance was measured with qPCR before and after DNA reextraction and compared using the Pearson correlation coefficient. White crosses denote significant correlations (p < 0.05).

**Figure S4: Metagenome abundance is also impacted by antibiotics.**

Principal coordinates plot of Bray-Cutis dissimilarity calculated for TPM-normalized gene abundance. PERMANOVA detected significant differences (p < 0.05) between treatment groups and experiment phases.

**Figure S5: Correlation analysis with 16S amplicon sequence abundance produces similar results to the shotgun abundance correlations in Figure 4.**

1. Heatmaps of Pearson correlation coefficients for the abundance of (**A**) *Bifidobacterium* and (**B**) *Fusobacterium* at the genus level. Axis labels denote the source of the data (qPCR or amplicon) as well as the type of abundance (rel = relative abundance; obs = observed abundance; clr = centered-log ratio, uL = gene copy number). Labels containing X’s denote multiplication (e.g. amplicon relative abundance * qPCR 16S gene abundance). White crosses denote significant correlations (p < 0.05) after adjustment at 5% FDR. Correlations were performed using amplicon sequence data generated from the same fecal samples as in Figure 4.

**Figure S6: Bristol stool scores increase as a result of antibiotic treatment.** Line graph of Bristol scores for the subset of fecal samples used in qPCR experiments. Colors denote treatment group averages and ribbons denote 95% confidence intervals in each experiment phase. Significance is denoted by number of asterisks and corresponds to p < 0.05 (*), p < 0.01 (**), and p < 0.001 (***). Brackets depict a change between experiment phases with respect to the Pre-Treatment phase, while asterisks without brackets depict group-phase interactions.
